# An Acoustic Device for Ultra High-Speed Quantification of Cell Strain During Cell–Microbubble Interaction

**DOI:** 10.1021/acsbiomaterials.3c00757

**Published:** 2023-09-25

**Authors:** Oliver Pattinson, Sara B. Keller, Nicholas D. Evans, Fabrice Pierron, Dario Carugo

**Affiliations:** † Faculty of Engineering and Physical Sciences, University of Southampton, University Road, Southampton SO17 1BJ, United Kingdom; ‡ Department of Engineering Science, University of Oxford, Old Road, Headington, Oxford OX3 7LD, U.K.; ¶ Nuffield Department of Orthopaedics, Rheumatology and Musculoskeletal Sciences (NDORMS), University of Oxford, Old Road, Headington, Oxford OX3 7LD, United Kingdom

**Keywords:** microbubbles, acoustic device, ultrasound, cell strain, ultra high-speed imaging, digital image correlation

## Abstract

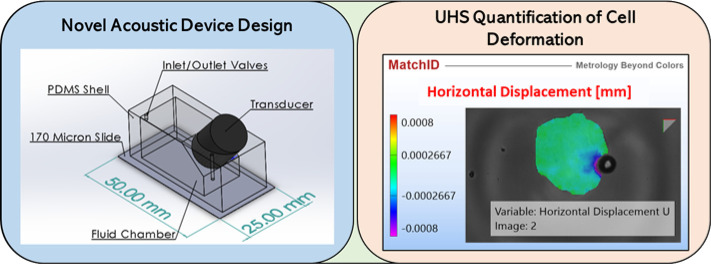

Microbubbles utilize high-frequency oscillations under ultrasound stimulation to induce a range of therapeutic effects in cells, often through mechanical stimulation and permeabilization of cells. One of the largest challenges remaining in the field is the characterization of interactions between cells and microbubbles at therapeutically relevant frequencies. Technical limitations, such as employing sufficient frame rates and obtaining sufficient image resolution, restrict the quantification of the cell’s mechanical response to oscillating microbubbles. Here, a novel methodology was developed to address many of these limitations and improve the image resolution of cell–microbubble interactions at high frame rates. A compact acoustic device was designed to house cells and microbubbles as well as a therapeutically relevant acoustic field while being compatible with a Shimadzu HPV-X camera. Cell viability tests confirmed the successful culture and proliferation of cells, and the attachment of DSPC- and cationic DSEPC-microbubbles to osteosarcoma cells was quantified. Microbubble oscillation was observed within the device at a frame rate of 5 million FPS, confirming suitable acoustic field generation and ultra high-speed image capture. High spatial resolution in these images revealed observable deformation in cells following microbubble oscillation and supported the first use of digital image correlation for strain quantification in a single cell. The novel acoustic device provided a simple, effective method for improving the spatial resolution of cell–microbubble interaction images, presenting the opportunity to develop an understanding of the mechanisms driving the therapeutic effects of oscillating microbubbles upon ultrasound exposure.

## 1 Introduction

Ultrasound waves can induce the cavitation of gas-filled microbubbles suspended in a liquid. The resulting oscillations in microbubble volume can perturb nearby structures through direct contact or manipulation of the surrounding fluid.^1,2^ Techniques combining ultrasound and microbubbles have been exploited for a range of applications in medicine and biology. For example, microbubble cavitation can inflict physical damage for tissue ablation,^3^ induce temporary or permanent membrane disruption in cells,^4^ or generate openings in intercellular junctions within biological barriers.^5^ These effects can be exploited for enhanced drug and gene delivery^6,7^ or for stimulating cellular transduction pathways.^8^ Currently, microbubbles are being investigated in a number of clinical trials exploring these applications, as they present the opportunity to enhance a range of current therapies in a nonharmful and minimally invasive fashion.

Despite progress in the application and development of ultrasound-responsive microbubbles, the fundamental interactions between these agents, the target cells and the surrounding medium are difficult to define accurately, mainly due to the high frequency of microbubble oscillation.^9^ As a result, in the majority of studies, aspects such as the mechanism of energy transfer between microbubbles and tissue, mechanical cell response, and immediate biological effects are not suitably captured and are often not defined. Commonly employed methodologies for detecting therapeutically relevant effects of cell–microbubble interaction often have low temporal resolution (in the range of seconds after US treatment^10^) when compared to the submicrosecond time scales at which microbubble oscillation occurs. A number of studies have successfully employed higher temporal resolution methods, i.e., from high-frequency fluorescence imaging in the microsecond range^11^ to imaging methods with interframe times as low as 40 ns.^5^ These studies were among the first demonstrating that optical methods can be utilized to reveal changes within the structure of the microbubble and cell at small time scales.^12,13^ However, they still lack precise quantification of the cell–microbubble interaction mechanisms.

The frequency of ultrasound used to induce microbubble cavitation is on the order of megahertz (typically in the range of 0.5–2 MHz). Therefore, according to the Nyquist criterion, an optical imaging method required to resolve and reconstruct the oscillation cycle of a microbubble must have a frame rate of at least two million frames per second (FPS) for a 1 MHz signal frequency.^14^ Ultra high-speed (UHS) imaging, encompassing imaging methods at >1 million FPS, was first used in this manner to track the oscillation response of microbubbles alone. An array of microbubble dynamics were thus observed using optical techniques for the first time,^15^ including microbubble formulation-dependent oscillatory behaviors,^16^ high-velocity microjetting,^17^ and nonspherical oscillations.^18^ These studies revealed how microbubble cavitation could be enhanced or controlled through experimental design and provided evidence for physical phenomena (such as fluid jetting) that may cause tissue permeabilization. Through these findings, UHS imaging influenced the advancement of therapeutic studies in the early 2000s.

Following advances in the imaging technologies available, it became possible to study the interaction between microbubbles and biological structures at a high temporal resolution. Previous *in vitro* studies in this area have observed microbubble-induced deformation and morphological changes in cells,^12,19^ as well as larger-scale deformation in tissue structures such as blood clots.^20^ Despite these studies being among the first to observe cell deformation and strain following microbubble oscillation, the imaging methods used could not provide the spatial resolution required to support the quantification of these mechanical responses at the single-cell level. More recently, studies have combined UHS imaging with techniques that can track therapeutically relevant outcomes at a lower temporal resolution. Cell membrane permeabilization (or sonoporation)^21^ or the opening of intercellular junctions^5^ could be observed and characterized in cells, alongside UHS imaging of cell–microbubble interaction. Results from these studies highlighted the complexity and heterogeneity in these interactions and subsequent cellular responses, supporting the need for analytical methods with greater spatial resolution that can resolve these phenomena quantitatively.

To develop a system that can quantify the mechanical response of cells at the relevant temporal frequency, we addressed the methodological approach for studying microbubble-cell interactions. Previous work in this area has employed different types of cell culture chambers, including commercial cell flasks that allow for imaging of microbubble-cell interactions but are not ideal for ultrasound stimulation purposes, due to their material and geometrical properties.^22,23^ Previous methods often involve placing the cell culture chamber within a water tank, which allows for optical transparency and the generation of a well-defined acoustic field. However, this approach is incompatible with some higher-resolution and/or quantitative imaging techniques due to the large distances between microscope optics and the light source. Other studies developed custom miniaturized devices to fit individual imaging requirements,^24,25^ but these systems are not compatible with concurrent bright-field imaging due to the presence of opaque components.

The experimental approach adopted in the present study was informed by the imaging methodology used by Seghir and Pierron.^26^ This method supported the use of digital image correlation (DIC), an experimental mechanics technique used to resolve high strain rate deformations in materials, which has also been applied in some biological studies although at lower imaging frequencies.^27,28^ Overall, the design criteria for the developed apparatus included the need to support cell culture within a fluid chamber that could allow for the microbubble interaction with cells. In addition, the device had to support a reliable and tunable acoustic field that replicated interaction dynamics similar to those in therapeutic and clinical studies. Finally, and most specific to this project, was the requirement to be compatible with an ultra high-speed imaging methodology which involved the use of a Shimadzu HPV-X camera and an inverted microscope. This translated to the requirement for a compact design with minimal working distances between the microscope objective and condenser to provide sufficient contrast and spatial resolution for DIC analysis.

In this article, the development and implementation of a novel acoustic device that meets the above-defined criteria are reported. This device facilitates high-resolution imaging of the cell–microbubble interaction at high frequency and subsequent quantification of cell deformation, which can be applied to begin addressing the mechanistic knowledge gaps in therapeutic microbubble studies.

## 2 Materials and Methods

### 2.1 Ultra High-Speed Imaging Set-up

**Figure 1:**
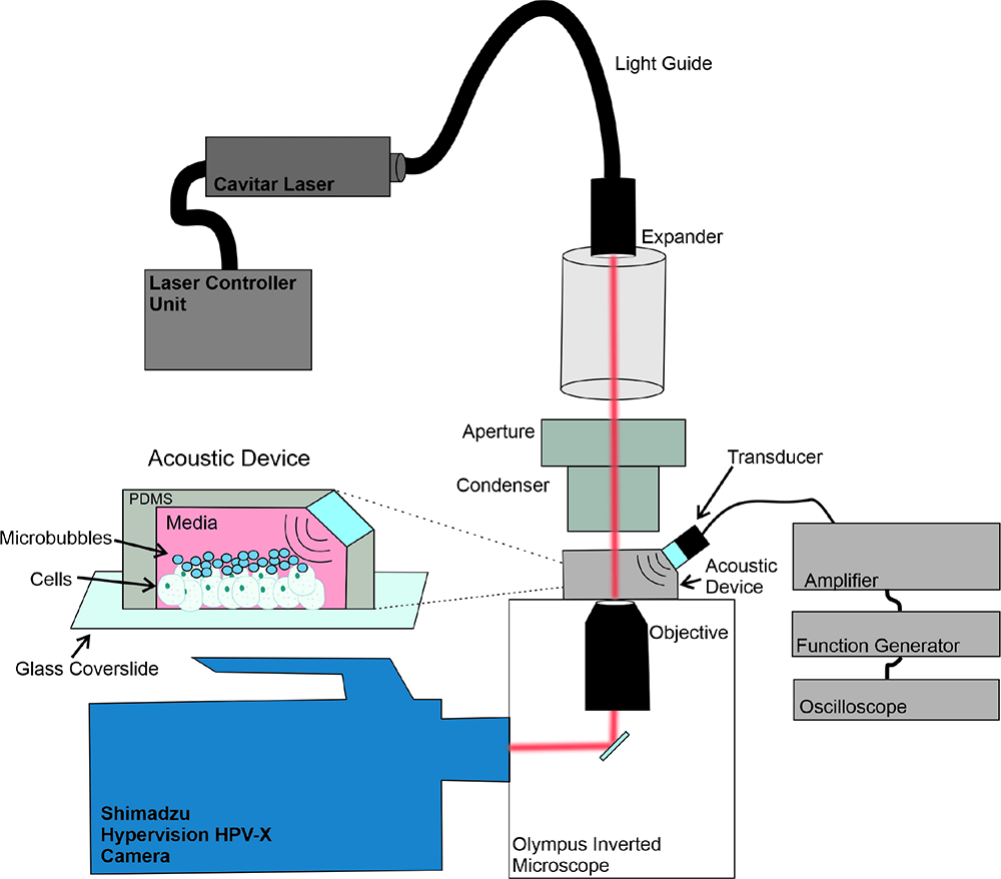
Diagram of the experimental system. Ultra high-speed images are obtained with a Hypervision HPV-X camera, and the illumination is provided by a Cavitar pulsed laser. The ultrasound field is generated by a 1 MHz piezoelectric transducer driven by a power amplifier, which is fed from a signal generator. The enlarged view of the acoustic device shows the acoustic stimulation of the cells and microbubbles.

To obtain sufficient temporal and spatial resolution to study cell-microbubble interactions using the Hypervision HPV-X camera (Shimadzu, Kyoto, Japan), an optical imaging system was designed to maximize the light available for the sample and the magnification during image capture. This system is displayed in Figure 1. The ultrahigh speed camera, capable of imaging up to 10 million frames per second, was coupled to an inverted Olympus IX 71 microscope (Olympus, Tokyo, Japan) equipped with objective lenses up to a maximum magnification of 80 ×. The camera uses FTCMOS technology and can record 128 frames of resolution 400 × 250 pixels.^29^ A Cavilux pulsed diode laser (Cavitar LTD, Tampere, Finland) was used to illuminate the sample. The laser has a power of 400 W, producing a red-light wavelength of 640 nm with a minimum pulse length of 10 ns. A control unit allows for variation of the pulse length to support the camera’s frame rate and optimize the lighting conditions. For the capture of cell–microbubble interactions under ultrasound exposure, frame rates of 5 million frames per second were used (as imaging at 10 million frames per second only captures half the pixels and sacrifices spatial resolution), with laser pulse lengths of 50 ns using an 80× objective. A signal generator was used to trigger the camera, to ensure synchronization between the acoustic field and light exposure for image acquisition.

### 2.2 Acoustic Device Manufacture

The design of the acoustic device was informed by three main factors; (i) the ability to support cell culture, (ii) the ability to house and maintain a reliable and suitable acoustic field, and (iii) the compatibility with the imaging setup described above. A design choice was made to create a miniature liquid-filled chamber for cell culture and acoustic field generation with a total height of less than 2 cm to fit between the microscope’s objective and condenser within the working distance. A thin glass substrate was used to culture the cells while enabling high-resolution imaging. Finally, polydimethylsiloxane (PDMS) was used for a moldable, acoustically compatible manifold which could adhere to glass and remain optically transparent. A secondary criterion for this manifold was an insert for the source of the acoustic field, which must be secure but also designed to maintain a clear optical path through the device. A transducer was custom-designed and supplied by Precision Acoustics (Dorchester, UK), with requirements of compact size and compatible frequency range around which the device manifold was designed. The transducer had a center frequency of 1 MHz, with a flat surface and an active element size of 10 mm and a housing size of 13.55 mm. This device design also supported the further analysis of cells postultrasound exposure. For example, cells could be collected from the device using traditional cell culture techniques and analyzed using flow cytometry or other analysis methods.

PDMS was prepared by combining PDMS precursor and curing agent (Sylgard 184, Farnell, Leeds, UK) at a 10:1 weight ratio, which were then mixed and subsequently degassed in a vacuum chamber. A mold was 3D printed out of poly(lactic acid) (PLA) using an Ultimaker S5 printer with 100% infill and a 6 mm layer height. The mold was filled with liquid PDMS and this was allowed to cure for 48–72 h. The solidified PDMS manifold was plasma bonded to a 170 μm thick (75 × 25 mm) glass cover-slide (Logitech Limited UK, Glasgow, UK) using a Zepto plasma surface treatment machine (Diener Electronic + CO KG, Ebhausen, Germany). Specifically, PDMS and glass were exposed to oxygen plasma for 30 s and then bonded together. Following bonding, the device was kept on a hot plate at 80 °C for 30 min before use.

Numerical simulations were performed using COMSOL Multiphysics 5.5 to optimize the device architecture based on the acoustic field properties within the fluid chamber. For a 2D model of the device’s cross-section, a minimum mesh size of 500 nm was used for the entire geometry. For a 3D model of the entire device, a minimum mesh size of 75 and 500 μm were employed for the glass slide and fluid layer, respectively. The ’Pressure Acoustics, Frequency Domain’ module was employed to determine the acoustic pressure distribution within the model. A 1 MHz ultrasound source was added with an arbitrary magnitude from the surface of the transducer. The COMSOL material library data for water, silica glass, and PDMS were used to model the fluid, cover-slide, and chamber manifold, respectively. The acoustic impedance of the materials was set to 1.48 MRayls for the fluid,^30^ 1.05 MRayls for the PDMS,^31^ and 13.0 MRayls for the glass substrate.^32^ Independent parameters including the ultrasound frequency, transducer’s inclination angle, transducer’s focal length, and thickness of the PDMS opposite the transducer inlet were also defined. It should be noted that exact acoustic pressure values cannot be accurately inferred from these simulations; hence, pressure values are normalized with respect to the maximum pressure and are reported on a scale from 0 to 1.

### 2.3 Experimental Characterization of the Acoustic Field

The acoustic pressure field within the device was characterized using hydrophone measurements in a water tank apparatus. The generated acoustic pressure was first calibrated over a range of driving voltages without the glass slide present, before a fiber optic hydrophone (Precision Acoustics, Dorchester, UK) was inserted through a hole made in the side-wall of the PDMS manifold opposite the transducer inlet. A scan in the *x*- and *z*-directions was performed to identify the focal point (i.e., the point of maximum pressure). A planar scan was then performed just above the glass surface, covering a region of 15 mm × 8 mm in the *x-z* plane around the focal point of the pressure field. The acoustic pressure field was quantified at a frequency of 1 MHz, with a driving voltage of 100 Vpp over 10 cycles.

### 2.4 Microbubble Production and Characterization

Microbubbles were prepared via two-stage sonication, adapted from a previously reported method.^33^ This method is known to produce microbubble suspensions with high concentration but large size dispersity^34^ and was selected as a suitable technique to rapidly produce microbubbles in this study. 1,2-Distearoyl-*sn*-glycero-3-phosphatidylcholine (DSPC) and polyoxyethylene (40) stearate (PEG40s) (Merck, Darmstadt, Germany) dissolved in chloroform were mixed inside 20 mL glass vials at a molar ratio of 9:0.5 for DSPC-microbubbles. Perforated parafilm was used to seal the vials, which were left for 24 h to allow the chloroform to evaporate, leaving a dry lipid film. The lipid films were rehydrated with 2.5 mL of Dulbecco’s Phosphate Buffered Saline (DPBS), while heated past the transition temperature of DSPC (55.6 °C) to 90 °C. This lipid dispersion was continuously stirred with a stir bar at 700 rpm on a magnetic hot plate stirrer for 45 min. The stir bar was removed and, a 20 kHz sonicator probe (Fisherbrand Model 120 Sonic Dismembrator, probe diameter 3 mm, maximum power 120 W, Fisher Scientific, Leicestershire, UK) was submerged near the base of the vial for a first sonication of 150 s at an amplitude of 40%. This was followed by a second sonication at the liquid–air interface for 30 s at 70% amplitude, which resulted in the formation of a microbubble suspension. The latter was then transferred to a freezer for 5 min to lower the temperature before microbubbles were stored in a fridge at 5 °C for use.

Prior to the chloroform evaporation stage described earlier, in some experiments, the cationic phospholipid 1,2-distearoyl-*sn*-glycero-3-ethylphosphatidylcholine (DSEPC) (Merck, Darmstadt, Germany) was added at a molar ratio of 9:0.5:2 (DSPC:PEG40s:DSEPC) to produce electrostatically charged DSEPC-microbubbles. Both microbubble formulations were characterized for their size and electrical charge (Supplementary Figure S1). The microbubble size distribution was measured from the analysis of microscopy images of microbubbles within a Neubauer hemocytometer based on the method described by Sennoga et al.^35^ A custom built ImageJ script was designed to perform the final sizing stage. Optical images were analyzed, and microbubbles were isolated from the background so that the ’Analyze Particles’ function could be applied which calculated the diameter of each microbubble. The microbubble zeta potential (i.e., a measure of electrical charge) was quantified by using a dynamic light scattering (DLS) apparatus. To perform this, a small sample of the microbubble suspension was diluted in distilled water and placed within a ZetaSizer Ultra (Malvern Panalytical Ltd., Worcester, UK) machine, resulting in a positive zeta potential of about 30 mV for DSEPC-microbubbles, compared to the slightly negative zeta potential of DSPC-microbubbles at around −5 mV.

### 2.5 Microbubble-Cell Attachment

Targeting of microbubbles to cells has been shown to improve microbubble imaging applications^36^ and is hence studied extensively within the therapeutic field.^37^ Successful targeting is desirable during *in vitro* experiments and must be supported within the designed acoustic device. In this study, targeting was verified by determining the attachment of microbubbles to cells following the inversion of a cell culture dish, in which untargeted microbubbles would float away from the cell surface. Two types of osteosarcoma cells, MG-63 and SaOs-2 (purchased from the UK Health Security and supplied by the European Collection of Authenticated Cell Cultures (ECACC)), were seeded at a density of 20 000 cells per cm^2^ in sealable culture dishes (μ-dish 35 mm, Ibidi GmbH, Grafelfing, Germany), i.e., a cost-effective, bulk testing system that could be used to control and verify cell–microbubble attachment before the acoustic device was employed. Following these experiments, cells were seeded under the same conditions in the manufactured acoustic devices detailed above. MG-63 cells were cultured in DMEM cell medium (Dulbecco’s Modified Eagle’s Medium, Lonza Group Ltd., Basel, Switzerland) supplemented with 10% v/v fetal bovine serum (FBS) and 100 μg/mL penicillin/streptomycin (P/S). SaOs-2 cells were cultured in αMEM cell medium (minimum essential medium - Alpha Eagle, Lonza Group Ltd., Basel Switzerland) supplemented with 10% v/v FBS and 100 μg/mL P/S.

After 24 h in a 5% CO_2_ incubator, the media was removed and the cells were washed three times with DMEM. Both the μ-dishes and acoustic devices were filled with 8 mL of plain DMEM supplemented with 5% v/v microbubble suspension and sealed. Both DSPC and DSEPC-microbubbles were investigated, with both cell types as well as a control group without cells. The dishes and devices were inverted for 5 min to induce contact between cells and microbubbles and were then reinverted to image the cell culture surface with an EVOS XL Core inverted microscope (Thermo Fisher Scientific, Waltham, MA, USA) using a 20× objective. Three images were taken per n number at random positions within the cell culture, at times 0, 2, 5, 10, 30, and 60 min after inversion. Images were processed using ImageJ in order to determine the number of attached microbubbles using a macro that applied the ’analyze particles’ function to count microbubbles in the image. To test the strength of attachment, following inversion, the cell–microbubble mixture was gently washed three times using DMEM for both microbubble and cell types, with images taken before and after the washing process. The same image processing steps were performed to quantify the number of microbubbles that remained attached to cells after washing, which highlighted whether the attachment could persist following low levels of shear flow.

### 2.6 Cell Viability Assessment

To assess whether the acoustic device could support reliable cell culture, a viability assay was performed on cells cultured within the device. MG-63 cells were seeded at densities of 10,000 cells per cm^2^ and cultured in a 5% CO_2_ incubator for up to 72 h. At three time points, the media was removed and the cells were washed with DPBS. Calcein AM cell labeling dye (Fisher Scientific, Leicestershire, UK) was used to stain the live cells. One milliliter of 4 μM Calcein AM solution in Hanks Balanced Salt Solution was added to the device and was then left to incubate at 37°C for 30 min. Live cells were subsequently imaged using fluorescence microscopy with an EVOS M500 microscope in the GFP channel (excitation/emission wavelengths of 470/525 nm) at 20× magnification. Successful cell culture was validated by the presence of fluorescence staining within the cells, indicating viability as well as propagation of the number of cells producing a fluorescent signal to indicate cell growth and proliferation.

### 2.7 Ultrasound Stimulation of Microbubbles in the Device

To reduce strain on the transducer while increasing the generated acoustic pressure, a pulsed ultrasound regime was adopted. This stimulation regime is also consistent with most of the therapeutic applications of ultrasound in conjunction with microbubbles. A HS3 Handyscope (TiePie, Sneek, The Netherlands) was used to generate the desired pulse function. A pulse repetition frequency (PRF) of 1000 Hz and a duty cycle of 30% was used in this study. Each pulse triggered a TG2000 function generator (Aim-TTi, Cambridgeshire, UK) to produce a 1 MHz pulsed sine wave. This wave was amplified before being delivered to the transducer to generate a 1 MHz ultrasound wave. For use with the HPV-X camera, the same signal used to trigger the signal generator was employed to trigger the camera to ensure that imaging took place during the appropriate ultrasound stimulation window. The transducer was inserted into a side port of the acoustic device, which had been previously seeded with MG-63 cells and incubated with microbubbles as described in section 2.5.

Ultrasound stimulation of microbubbles was confirmed by video capture of microbubble oscillation, using the ultra high-speed imaging system outlined in section 2.1. Frames from 5 million FPS videos, as triggered by the ultrasound stimulation described above, were then analyzed to identify changes in microbubble diameter. The frames were processed using an edge detection algorithm, developed by Trujillo-Pino et al., measuring the change in microbubble size at subpixel accuracy.^38^ Oscillations of microbubbles were captured under an array of different imaging and acoustic settings to validate the versatility of the imaging method.

### 2.8 Measuring Cell Deformation from Microbubble Stimulation

Cellular deformation was confirmed by two methods. First, gray-level variation was examined using ImageJ software over selected regions of interest to determine displacement throughout the UHS video. Second, digital image correlation (DIC) was performed using MatchID software (MatchID 2D, 2021.2.2) to determine deformation and strain within the cells over the length of the video, using a natural speckle pattern observed on the cell surface. The first frame is used as the DIC reference image, and then subsequent frames are processed using a zero-normalized sum of squared differences (ZNSSD) correlation with an affine shape function. Colored plots reveal how the deformation changes throughout the cell over the length of the video. At the same time, specific numerical data can also be extracted and used to perform a quantitative analysis of the cell deformation behavior.

## 3 Results

### 3.1 Acoustic Device Design, Manufacture, and Characterization

**Figure 2:**
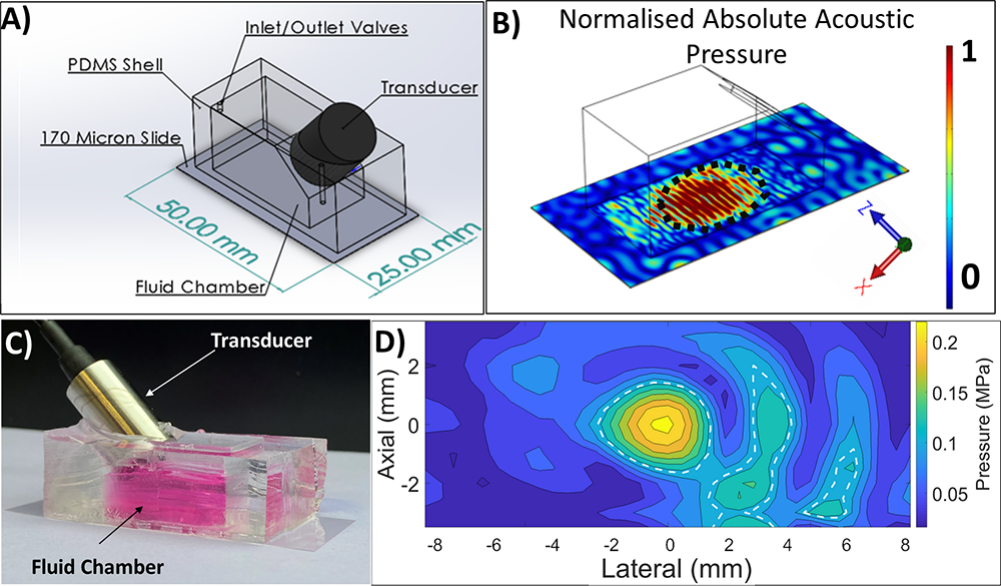
Acoustic device design, manufacture, and acoustic characterization. (A) CAD diagram of the acoustic device, comprising an ultrasound transducer inserted through a PDMS manifold bonded to a glass cover-slide, with dimensions to scale (reference geometry in Supplementary Figure S2). (B) The simulated spatial distribution of the normalized acoustic pressure field over the glass cover-slide surface (i.e., where cells are seeded) was determined from three-dimensional numerical simulations. The dotted line indicates a region with pressures above 70% of the peak pressure. (C) Photograph of the manufactured acoustic device, with a half-filled fluid chamber and 1 MHz transducer inserted through the PDMS manifold. (D) Spatial distribution of the measured acoustic pressure within the device over the glass slide, obtained using a fiber optic hydrophone with the transducer inserted at the right side of the device (consistent with the orientation shown in B). Regions delimited by the white dashed lines indicate pressures within −3 dB of the peak.

The finalized CAD design generated by applying the design criteria described in section 2.2 is shown in Figure 2a. Specifically, the fluid chamber is of a suitable size to enable visualization of the cell culture through the device during optical microscopy and to provide a reservoir of nutrients for cell culture. The architecture of the PDMS manifold allows the insertion of an ultrasound transducer to generate a therapeutically relevant ultrasound field. This insertion is at an angle that directs the acoustic field to the cell culture surface, which is crucial to meeting the criteria of a clear optical path. This facilitates unobstructed imaging of the point in the device where the acoustic field meets the cell–microbubble interactions. Finally, the dimensions of the glass substrate allow the device to fit on a standard microscope stage and are also compatible with high-resolution microscopy.

The acoustic field within the device was simulated numerically prior to its manufacture to inform the selection of appropriate design parameters and gain a more pervasive understanding of the ultrasound exposure conditions within the device. The 3D simulation indicated a region of high acoustic pressure at the glass substrate within an area of approximately 167 mm^2^ in which the pressure was greater than 70% of the simulated peak pressure (Figure 2b). Despite pressure nodes and antinodes being visible in both simulations, a consistent region of high pressure is observed within the imaging area, which is located at the surface where cell–microbubble interactions will occur. Profiles in the *x* and *y*-direction and a 2D cross-section of the simulated pressure along the glass slide support this 3D simulation and reveal similar nodes in the vertical direction (Supplementary Figure S3).

Figure 2c is a photograph of the final manufactured device. It shows that PDMS is a suitable material to manufacture a manifold that is sufficiently robust mechanically as well as optically transparent, i.e., to allow both the visual detection of air pockets during priming and bright-field microscopy imaging. It also shows that the bonding technique used creates a watertight fluid chamber capable of holding the required amount of cell culture media. Finally, it further confirms that the device supports the insertion of an ultrasound transducer of suitable specifications while being held on a commercial microscopy cover-slide. In the photograph, the fluid chamber is only half filled, and for application, it will be completely filled and the transducer’s active surface will be in direct contact with the liquid medium.

The experimentally measured acoustic pressure field had some comparable characteristics to the simulated field, with a central peak pressure at the focal point and a significant reduction in pressure levels toward the outer edges of the fluid chamber. The maximum acoustic pressure was found to be about 220 kPa, and there was a −3 dB bandwidth of about 4 mm in the *x*-direction and 3 mm in the *z*-direction. Two other regions closer to the transducer (right) were seen, which also fell within 3 dB of the maximum pressure, while no similar regions were observed on the opposite side (left) of the peak pressure. The pressure nodes found in the simulated pressure field were less apparent in the experimentally measured field.

In a complementary series of simulations, the effect of a number of design parameters on the acoustic pressure distribution along the cell culture surface (i.e., the liquid-glass substrate interface) was examined. These simulations showed that the PDMS thickness did not alter the acoustic pressure field significantly while the transducer’s driving frequency, inclination angle and focal length created varied distributions in the acoustic pressure along the glass slide (Supplementary Figure S4). This meant the PDMS thickness did not need to be finely controlled during experiments while an ultrasound driving frequency of 1 MHz, a transducer inclination angle of 50°, and a focal length of 12 mm were chosen to maximize the area of peak acoustic pressure and limit spatial variations in the acoustic pressure distribution.

### 3.2 Cell Culture in the Acoustic Device

**Figure 3:**
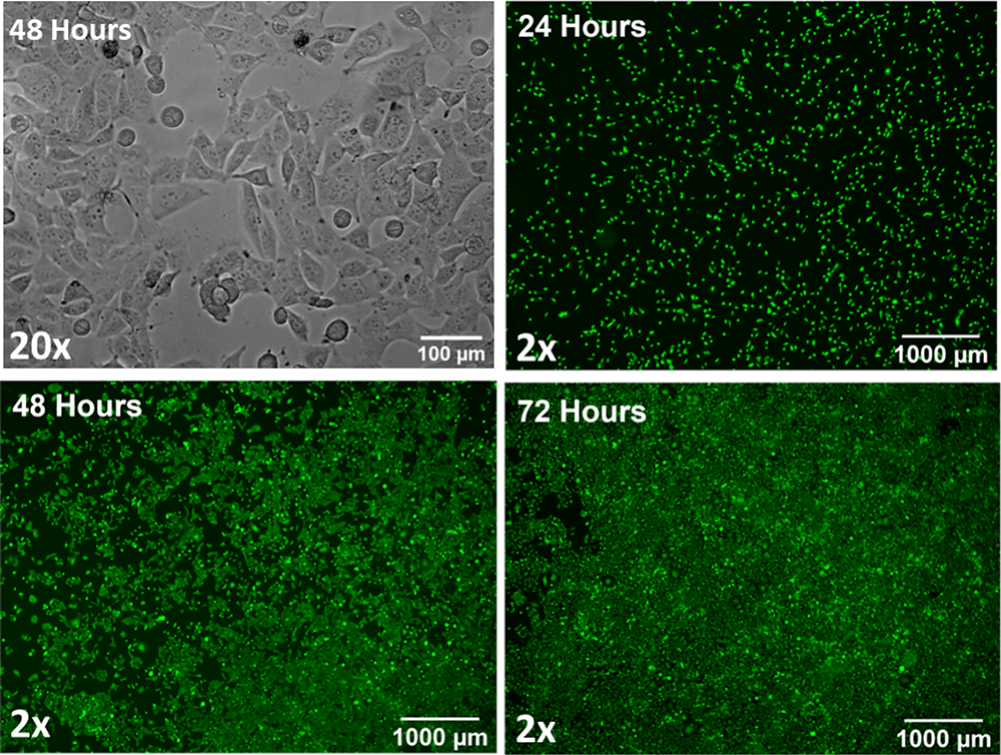
MG-63 cells cultured in the acoustic device for 24, 48, and 72 h. Cells were imaged with a 20× objective for the bright-field images and a 2× objective for the fluorescence images. Cells were stained using Calcein AM fluorescent dye, showing viable, live cells in green. Imaging was carried out using an EVOS M500 microscope in the transmitted light and GFP channel settings.

Cells were seeded in the manufactured devices, and a fluorescent viability assay was employed to ensure the device could support adequate cell culture. MG-63 cells attached and proliferated on the glass substrate within the devices as seen in Figure 3. The phase contrast image in the first frame (20 ×) shows that after 48 h, cells have adhered and display a healthy morphology. At this time point, cells are also subconfluent and can be imaged clearly at higher magnification. Cells survived over longer time scales and proliferated over a period of 72 h, as evident by an increase in the number of calcein-stained cells imaged at lower magnification (2×).

### 3.3 Microbubble-Cell Attachment

**Figure 4:**
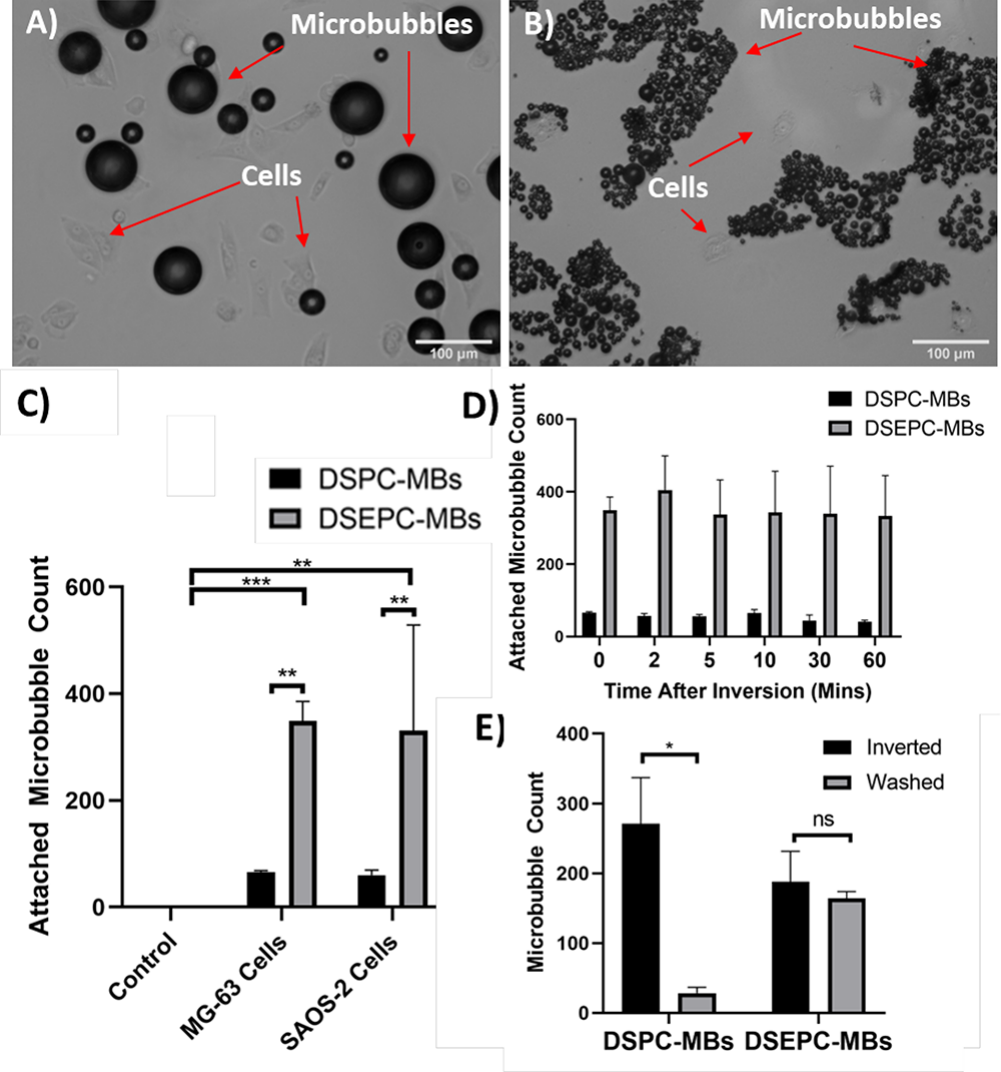
Microbubble-cell attachment observed in the acoustic device. (A) Attachment between MG-63 cells and uncharged DSPC-microbubbles in the acoustic device and (B) attachment between MG-63 cells and cationic DSEPC-microbubbles in the acoustic device. Quantitative analysis of microbubble attachment was carried out in an Ibidi μ-dish. (C) Following 5 min of inversion, the number of uncharged DSPC- and charged DSEPC-microbubbles attached to MG-63 and SaOs-2 cells, compared to an empty dish (control). (D) Number of microbubbles that remained attached to MG-63 cells for up to 1 h following inversion for both bubble types (*p* < 0.05 for all comparisons between DSPC- and DSEPC-microbubbles). (E) shows the effect of washing both types of microbubbles attached to MG-63 cells with DMEM, following reinversion (* = *p* < 0.05, ** = *p* < 0.01, *** = *p* < 0.001, **** = *p* < 0.0001).

Following the addition of microbubble formulations to cell-containing acoustic devices, interactions between microbubbles and cells were visible at the glass substrate surface (Figure 4a,b). Although the microbubbles are buoyant, for both neutral and charged microbubble formulations, many microbubbles remained within the imaging plane, indicating that there is attachment between the microbubbles and either the cells or the glass surface. It is also observed that a greater number and wider size distribution of microbubbles remain attached for the cationic DSEPC formulation. Smaller DSEPC-microbubbles can attach to cells and in much greater numbers, whereas much larger DSPC microbubbles attach to cells with radii greater than those of any DSEPC microbubble observed to become attached.

To determine quantitatively the interaction between charged and uncharged microbubbles and MG63 cells, microbubbles were added to cells growing in ibidi μ-dishes, and the dishes were inverted for 5 min to allow attachment. Microscopy images were then taken and microbubbles that remained associated with cells were counted, as described in the methods section. Significantly more charged microbubbles were found associated with cells as compared to uncharged microbubbles, in either MG63 or SaOs-2 cells (*p* < 0.01 for both cells, *n* = 3, see Figure 4c and Table 1). Notably, there was no detectable attachment of microbubbles to the surface of dishes that did not contain cells. For both microbubble formulations, the number of microbubbles that remained attached over an hour period remained consistent, as shown in Figure 4d and Table 2. In order to evaluate the strength of microbubble attachment, cells were washed with DMEM after dish reinversion. For neutral DSPC-microbubbles, the number of attached microbubbles reduced from 271 ± 66 to 28 ± 8 (*p* < 0.05) and for charged DSEPC-microbubbles, there was a small reduction from 188 ± 44 to 164 ± 10 (*p* > 0.05, *n* = 3 for all samples, see Figure 4e). These results indicate that different formulations of microbubbles could be reliably attached to cells for up to 1 h, with an attachment strong enough to resist low shear forces exerted during media washing.

**Table 1:** Mean Attachment of Microbubbles to Cells with Standard Deviations for Both Neutral and Cationic Microbubble Formulations (*n* = 3)

Cell type	DSPC-Microbubbles	DSEPC-Microbubbles
Control	0	0
MG-63	66 ± 2.6	349 ± 36.23
SaOs-2	60 ± 9.5	331 ± 197.2

**Table 2:** Mean Attachment of Microbubbles to Cells with Standard Deviations over a 60 min Period, Comparing Neutral and Cationic Microbubble Formulations (*n* = 3)

Microbubble	0 min	2 min	5 min	10 min	30 min	60 min
DSPC	66.0 ± 22	56.8 ± 5.4	55.5 ± 4.7	64.1 ± 8.4	42.3 ± 12.6	40.5 ± 3.9
DSEPC	348.1 ± 29.6	397.0 ± 77.0	327.4 ± 77.9	331.2 ± 92.8	322.5 ± 107.4	320.5 ± 91.0

### 3.4 Ultra High-Speed Microbubble Oscillation

**Figure 5:**
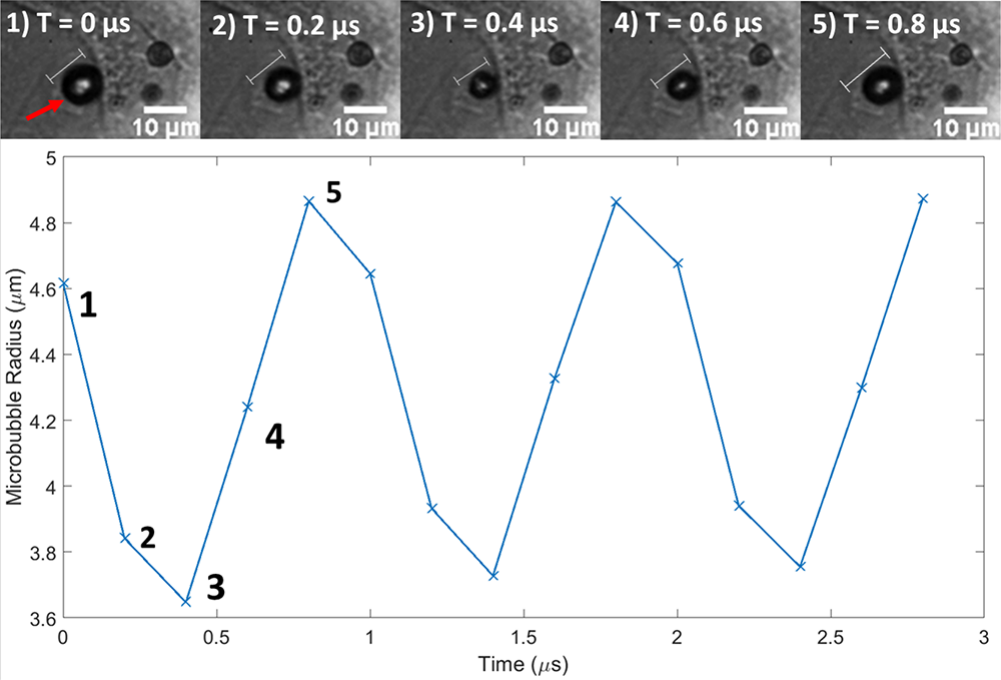
Video frames (top) and microbubble radius over time (bottom) showing microbubble oscillation within the acoustic device, captured at 5 million frames per second (with a Shimadzu camera) at 80× magnification. Images show DSPC-microbubbles attached to an MG-63 cell, and graphical data are representative of the change in the radius of the microbubble marked by the red arrow in the image. Scale bar: 10 μm.

Next, to determine the suitability of the acoustic device for inducing microbubble oscillation and measuring microbubble/cell dynamics, we stimulated the adherent microbubbles with pulsed ultrasound and imaged them concurrently at a frame rate of 5 million FPS. Sufficient light was available within the device using this method, which allowed us to resolve the microbubble and the cells, as can be seen in Figure 5. In 5 consecutive frames, over the course of 0.8 μs, volumetric oscillations of a microbubble of interest were visible, with an observable change in microbubble size between each individual frame 200 ns apart. The full video file is available in Supplementary Video S6. Using the edge detection method described above, it was possible to measure the diameter of the microbubble with respect to time over several ultrasound cycles, resulting in the radius vs time curve. The oscillation takes on a cyclic pattern with a frequency of 1 MHz, matching the ultrasound driving frequency with a maximal radial change of 1.22 μm (a percentage change of 25% from the mean radius). The results also highlight that the oscillation is stable over the 3 cycles represented; however, the full data set confirms this microbubble oscillates with the same amplitude for the entirety of the UHS video (see Supplementary Figure S5).

### 3.5 Ultra High-Speed Cell Deformation and Strain

**Figure 6:**
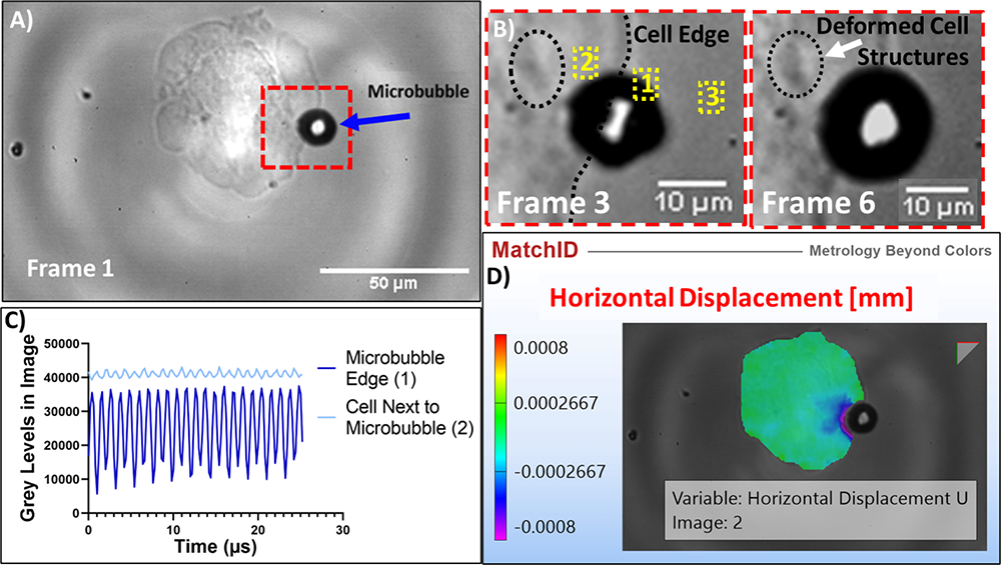
Visual results showing observable deformation in a cell interacting with an oscillating microbubble and quantitative analysis from the image and through DIC. (A) Reference frame for the video showing a DSEPC-microbubble interacting with an MG-63 cell, with the region of interest in the red box. (B) The magnified region of interest from the third and sixth frames of the UHS video shows cell structures (in black circle) which displace between image frames; note that this deformation is clearer in Supplementary Video S7. Three points of interest are highlighted in yellow. (C) Graphical results showing the gray level changes in the images over two of the points of interest over all 128 frames of the UHS video. Point 3 is taken as background gray level variation due to fluctuations in illumination, and was subtracted from the other values. (D) DIC frame produced using MatchID software, displaying the quantified spatial pattern of horizontal deformation throughout the cell via color plot.

Image analysis was performed to determine and subsequently quantify the deformation of nearby cells following the oscillation of the associated microbubbles. The UHS images collected by the HPV-X camera display clear evidence that the cells are deforming, in particular within the region of the cell that is closest to the oscillating microbubble, which can be seen by comparing the initial video frame (Figure 6a) and two frames taken 0.4 and 1 μs later respectively (Figure 6b). Observing the UHS video in its entirety makes the discerning cell deformation much clearer (Supplementary Video S7). This observable change can be quantified through the gray-level values of specific regions of interest within the images, which show oscillatory variations in areas of deformation (Figure 6c). The greatest amplitude of oscillation in gray-level is observed at the region encompassing the edge of the microbubble (Region 1), while a smaller oscillation of the same frequency is observed in the cell close to the microbubble (Region 2). These results reveal that deformation is observed visually in both the microbubble and the cell.

DIC results quantify the magnitude and spatial distribution of the visually observed deformation across the entirety of the cell, showing a concentration of horizontal deformation with a maximum value of 800 nm in the region directly adjacent to the microbubble (Figure 6d). This deformation is decreased at distances further from the microbubble, with the majority of the cell displaying a small magnitude of deformation within the range of 0–50 nm, which is caused by the noise of the camera. MatchID also produces an array of additional deformation and strain values because of the high level of resolution achieved with this technique. While Figure 6d displays the quantification of the cell deformation in the second frame of the UHS video only, the temporal change in deformation is seen to follow the oscillation of the microbubble when all of the UHS frames are analyzed (Supplementary Video S8).

## 4 Discussion

In this study, the design and manufacture of a novel acoustic device have been reported, and its compatibility with cell culture, acoustic stimulation of microbubbles, and quantification of the resulting cell deformation by DIC analysis has been demonstrated. The results reported in this paper indicate that the conceptualized device meets the postulated design criteria and supports the implementation of a novel method to determine the mechanical behavior of single cells upon exposure to ultrasound-activated microbubbles.

### 4.1 Device Design and Characterization

The compact design and small footprint of the developed acoustic device make it possible to perform ultrasound stimulation and image acquisition by using an optical microscope at high spatial resolution. In comparison to other studies that utilized acoustic devices for ultra high-speed imaging, the number of methodological challenges is greatly reduced. Studies that utilized the Brandaris-128 ultra high-speed camera,^5,19,22^ which produced much of the seminal work utilizing UHS imaging to study the behavior of microbubbles, often employed relatively large water tanks for ultrasound transmission in addition to a cell chamber (such as an OptiCell, Thermo Fisher Scientific Inc.). This resulted in limited optical magnification, potentially due to the large distances between either the objective lens or the condenser and the target cells, or the difficulty in providing sufficient illumination through the water tank from above. Equally, the Ultranac system - another similar UHS methodology which employs a smaller culture chamber immersed in a larger water tank to capture the behavior of ultrasound-stimulated microbubbles - is subject to the same constraints.^21,39,40^ A 40× optical magnification is the greatest used in these earlier studies, and despite both the Brandaris and Ultranac cameras being capable of imaging at greater frame rates than the imaging system employed here, neither study could temporally resolve the mechanical response of cells. It is expected that this is due to the low spatial resolution of the UHS images, with potentially insufficient contrast to accurately quantify the spatial changes in cell morphology. Conversely, the design of the acoustic device developed in the current study relied on the use of a 170 μm thick glass substrate for cell seeding and thus minimized the distance between the objective lens and cells, enabling imaging through higher magnification objectives. Moreover, the distance between the condenser, including the light source, and cells was also reduced to less than 5 cm. These characteristics of the device enabled imaging at greater magnification and resolution for a given camera. This resulted in greater pixel resolution for images of a single cell, enhancing the accuracy of image-based measuring techniques (as seen in Figures 5 and 6). Overall, findings presented in this paper indicate that the compact nature of the device successfully improves the image quality of UHS imaging when used to study cell–microbubble interactions. It should also be noted that as the device is fabricated by replica molding and the ultrasound source is reversibly coupled, multiple replicas of the device can be manufactured in relatively short time scales, making it suitable for high-throughput experimentation.

Alongside improved imaging conditions, a sufficient area of suitably high acoustic pressure located at the center of the device substrate (Figure 2) indicates that both cells and microbubbles were subject to therapeutically relevant acoustic stimulation conditions. The region found to exhibit acoustic pressures within 70% of the maximum pressure was considerably smaller than that predicted by the simulations. A reason for this could be the difficulty in obtaining a measurement precisely at the glass substrate using a hydrophone. The numerical simulations predict a reduction in the magnitude of acoustic pressure away from the glass substrate; therefore, the acoustic pressure field size and magnitude captured by the hydrophone are predicted to be under-estimations. Despite this, the size of the central peak of acoustic pressure has proven to be suitable for capturing individual cell–microbubble interactions. A limitation may be observed in the relatively steep reduction in the magnitude of acoustic pressure away from the central region of maximum pressure which would affect the dynamics of these interactions depending on the position within the device. In comparison to previously employed methods, the region of peak acoustic pressure recorded for this design is similar in size and shape to other compact device models,^25^ while it is about half the size of those typically generated in larger water tank systems using focused ultrasound.^10,22^ The measured magnitude of acoustic pressure within the device gives an accurate characterization of the acoustic stimulation felt by cells and microbubbles, which can be compared to the pressures employed in more traditional methodologies for therapeutic studies. In the literature, the pressure values reported to induce microbubble oscillation are within a wide range. In some studies, values as low as 25 kPa^41^ and 40 kPa^42^ are reported as a threshold for inducing microbubble oscillation. In contrast, much greater pressure levels, in the range of 2.0 to 2.5 MPa,^43,44^ and even up to 3.5 MPa,^45^ are also reportedly used for therapeutic applications of microbubbles. These values cover 2 orders of magnitude, and the magnitude recorded within this device falls approximately in the middle of this range. This confirms that the acoustic pressure generated is comparable to therapeutic studies and is expected to induce microbubble oscillation within the device. A peak magnitude of around 220 kPa was measured, which implies that microbubbles are likely to undergo stable (i.e., repeated) oscillations over multiple cycles. This is corroborated by the results shown in Figure 5. Stable microbubble oscillation has been known to be a product of lower acoustic pressures when compared to transient microbubble oscillation, often referred to as inertial cavitation.^46,47^ The study of these two types of oscillation (or cavitation) regime reveals lower threshold pressures for stable cavitation than transient cavitation, such as 200 kPa compared to 1.3 MPa,^48^ 250 kPa compared to 400 kPa,^49^ or 300 kPa compared to 450 kPa.^50^ This proves that the full scope of microbubble responses may not yet be obtainable with the current device design.

It is well-known that both regimes of cavitation are observed to play a role in therapeutic microbubble-based treatments,^7,19,51^ and so it is important to study both of them for their effect on cells. It is therefore a limitation of the device that the current design is restricted to inducing predominately stable microbubble oscillations. For the study of transient cavitation and its effects on cell deformation, it is likely that a more powerful transducer would be required, which may affect the design of the device. Equally, the spatial distribution of the acoustic pressure field within the device could also be improved to obtain a more uniform field as well as greater acoustic pressure magnitudes. This could be achieved in the future through the inclusion of absorbing material within the device to limit ultrasound wave reflections or by adjusting the transducer’s model and design. It is, however, our belief that the principles underpinning the device’s design could remain largely unchanged and that the methods presented in this study could be adjusted and employed to investigate the effects of transient cavitation, just as it has been demonstrated for stable cavitation. These limitations of the device, however, do not diminish the novelty and effectiveness of the reported design and techniques, as a range of microbubble oscillation amplitudes can be studied, as well as nonspherical stable oscillation patterns, and many different types of cell interaction conditions.

### 4.2 Cell Culture in the Acoustic Device

The result that cells could be cultured in the device for up to 72 h highlights that the acoustic device successfully meets a critical design criterion and facilitates cell proliferation (Figure 3). Previous techniques employed using culture chambers for cell–microbubble interaction studies have reported a culture time of 24 h^52,53^ and therefore the device reported here meets the current standards necessary for *in vitro* ultrasound stimulation and high-resolution imaging of cells.

### 4.3 Microbubble-Cell Attachment

Strategies to target microbubbles to cells have been shown to influence microbubble physicochemical characteristics,^54,55^ as well as their therapeutic outcomes both *in vitro*(22) and *in vivo*.^56^ The ability to induce and study targeted interactions between cells and microbubbles *in vitro* is an important device design criterion. It would also enable studying a wide range of cell–microbubble interaction conditions as well as quantifying the effects of cell-targeting specifically.

The finding that DSPC-microbubbles can form attachments with different osteosarcoma cell lines (Figure 4a) indicates that passive interactions between lipid-shelled microbubbles and bone cells occur under *in vitro* conditions. This means that we can employ a simple, effective method of inducing cell–microbubble interaction within our device, which places these interactions in the imaging plane of a high-resolution imaging system. Microbubble attachment to cells has been reported using other experimental systems, such as the OptiCell culture chambers,^22,57^ cover-slides without a coupled fluid chamber,^58^ or using suspended cells followed by flow cytometry.^59^ Each of these methods provides the opportunity to study aspects of microbubble-cell attachment yet is incompatible with the type of imaging proposed in this study. Other techniques of inducing interaction utilize acoustic radiation forces^60^ or fluid flow.^61^ These approaches enable the investigation of different modalities of attachment or interaction, but the dynamic nature of the process would introduce more complexities when implemented with ultra high-speed imaging. Therefore, the approach that was taken herein was deemed the most appropriate to validate the device’s capabilities.

Many studies have shown that interactions between microbubbles of different shell formulations and cells of different phenotypes occur, and hence, the interaction mechanisms quantified here are not notable in themselves. However, we have demonstrated a method to enhance the extent of microbubble interaction with cells based on the incorporation of the cationic phospholipid DSEPC into the microbubble shell (Figure 4b), which may facilitate ultra high-speed imaging of microbubble-cell interactions upon ultrasound exposure. Previous methods have demonstrated attachment using ligand–receptor techniques,^62,63^ electrostatically charged microbubbles, or a combination of both.^58,64^ Techniques based on ligand–receptor binding are seen as the most promising for therapeutic treatment due to their reliability and biocompatibility. In our results presented in Figure 4c, cell attachment was increased by a factor of 5 when using DSEPC compared to DSPC microbubbles. This supports targeting efficacies reported in previous studies, which are in the range of 5–8 times greater attachment for charged microbubbles.^58,64^ While Nomikou et al. and Zhou et al. reported a slight increase in attachment for biotinylated charged microbubbles compared to charged microbubbles, the significance is not comparable to the difference between charged and uncharged microbubbles. For the current type of mechanistic *in vitro* study, the use of specific ligands can be seen as an unnecessary and costly step. While microbubble attachment based on electrostatic interaction does not have the same *in vivo* potential due to coagulation and clotting caused by the interaction with other constituents in blood,^65^ it has many benefits for usage *in vitro*. The simplicity of an electrostatic attachment, compared to the more complex biological ligand–receptor method, makes it suitable to achieve fast, repeatable, and reliable microbubble binding to the cell membrane. It should also be noted, however, that the methodology reported in this study could be easily adapted to support other clinically applicable targeting methods if required. In future work, the device could also be employed to develop a novel understanding of the effects that targeting has on microbubble-induced cell mechanics.

### 4.4 Ultra High-Speed Cell Quantification of Cell–Microbubble Interactions

The observation that microbubble oscillation and cell deformation can be imaged in our system (Figures 5 and 6) indicates that the Shimadzu HPV-X is suitable for such studies. To our knowledge, this is the first report showing the use of this system in microbubble studies. Previous reports have employed similar camera models, such as the Shimadzu HPV-X2 camera, and successfully imaged microbubble oscillation,^66,67^ but these have not developed into quantitative studies exploring the mechanical response of cells. This may be due to increased gray level noise present in the HPV-X2 model,^68^ which makes high spatial resolution at high magnification difficult, or it could be a product of the imaging methodology and acoustic devices employed rather than the camera. Without quantification of the oscillation pattern of microbubbles, any new method cannot compete with previous techniques used to study microbubble cavitation, such as those based on rotating mirror high-speed cameras,^17,42^ image-converting high-speed cameras^21,40^ or acoustic monitoring techniques.^69,70^ In these earlier studies, changes in microbubble diameters within the range of 0.5–4 μm were observed, and the 1.2 μm radial oscillation captured in this device falls within this range. Herein, we have demonstrated the ability of the HPV-X camera to image at sufficient resolution to support accurate quantification of radial changes of a microbubble during stable cavitation to an extent that describes the entire oscillation response.

Our findings also demonstrate that the combined application of the HPV-X camera and the acoustic device provides sufficient spatial resolution to quantify the extent of microbubble-induced cell deformation at a resolution greater than that previously reported. To the best of our knowledge, this is the first study demonstrating high-frequency quantification of deformation across a single cell using DIC. The spatial resolution of deformation also supports further developments in quantifying the strain within cells, which is also generated through MatchID and DIC analysis. Deformation quantified in this study, both through gray level analysis and DIC, is observed to follow an oscillatory pattern at a frequency of 1 MHz (Figure 6), clearly evidencing that the oscillation of the microbubble drives the deformation of the cell. The localization of deformation to the area of the cell closest to the microbubble also indicates the correlation between microbubble interaction and the extent of deformation a cell experiences. As there is no deformation that follows an oscillatory pattern in areas near oscillating microbubbles without a cell present and in control videos where no microbubble is present at all, we can confirm that the measured deformations are true representations of microbubble-induced cell deformation. This demonstrates the novelty and potential impact of the designed device. The seminal work from the previous two decades, which employed UHS imaging to study cell–microbubble interactions, has had significantly lower levels of deformation quantification. Previously, studies which report measuring cell deformation following microbubble interaction have reported a single deformation that describes the response of the entire cell, rather than resolving it spatially.^12,71^ Other studies have implemented small levels of spatial resolution, describing the deformation of the cell localized to the microbubble oscillation, but again only report a single value of deformation, rather than a distribution.^72^ This makes a direct comparison with the deformation levels measured in this study more difficult. However, it can be seen from the results in Figure 6d that the deformations imposed on the cells are comparable to the magnitudes observed previously, with changes of around 800 nm measured in the current study, compared to ranges of 500 nm up to 2 μm in the literature. Further to this, a wide range of literature discusses the effect of observed deformation following UHS or slower imaging techniques, yet does not report quantified changes in cell deformation or mechanical response at any level.^10,20,40^

This considerably higher level of spatial resolution and deformation quantification has multiple advantages in the study of microbubble-based therapies. Since cell strain can feasibly be quantified using these methods, the study of its correlation with membrane permeabilization, activation of mechanotransductive pathways, and other therapeutically relevant phenomena can be evaluated to advance the understanding of therapeutic studies. Notably, the greater resolution obtained using the described methodology allows for new insights into the mechanical response of cells at high frequencies. It presents the opportunity to develop on the previously reported deformation values averaged across an entire cell mentioned above as well as quantifying deformation in studies where it is only qualitatively described. Spatial resolution at this frame rate is very often seen as difficult or even not possible to achieve due to the technological restrictions associated with ultra high-speed cameras, and hence the developed methodology addresses a common challenge in the field. As seen with microbubble-induced cell deformation, solving this challenge provides the opportunity for groundbreaking and novel findings in the area of ultrasound-mediated therapies and cell rheology.

The imaging method reported in this study suffers from some limitations relating to its spatial and temporal resolution. The camera used has a 5 million FPS upper limit without sacrificing the spatial resolution and has fewer pixels compared with previously used cameras. However, our findings prove that both these limits do not affect the ability to capture relevant cell and microbubble responses at a therapeutically relevant frequency of 1 MHz. Given its small size, the device could also be easily adapted for use with other cameras and techniques for cases where these limitations may restrict results. Therefore, this highlights that the presented device has many possible future applications with high magnification or high temporal rate imaging of samples under an acoustic field.

## 5 Conclusion

In this report, the design process, manufacturing, and evidence of application are presented for a novel acoustic device for the ultrasonic stimulation of cell–microbubble interactions. The compact nature of the design has been proven to optimize ultra high-speed microscopy of these interactions, while still producing a suitable acoustic field relevant to therapeutic microbubble applications. Example interactions have been demonstrated within the device and captured at frame rates of 5 million frames per second. This design, combined with ultra high-speed imaging and digital image correlation methodologies, has supported the first spatial quantification of cell deformation as a result of microbubble oscillation. This application provides evidence of the capabilities of this device, introducing the opportunity to bring new understanding and the necessary quantification for the study of the mechanical response of cells at high frequency. Future applications can utilize the developed device and methods to further explore correlations between parameters that dictate the interactions between cells and microbubbles, such as the size, number, and type of microbubbles, and the resulting deformation of cells.

## Supporting Information Available

Supporting file S1–S5: Microbubble and additional acoustic device characterization with reference geometry and COMSOL simulations and extended microbubble oscillation results to show the complete data set (PDF); Supporting Video S6: UHS video from Shimadzu HPV-X at 5 million FPS of microbubble oscillating in the presence of a cell (MPEG). Supporting Video S7: UHS video from Shimadzu HPV-X at 5 million FPS of microbubble oscillating in the presence of a cell (MPEG); Video S8: DIC video generated by Match-ID for horizontal deformation of an entire UHS video captured at 5 million FPS (MPEG) (ZIP)
