# Supplementary material for: An Acoustic Device for Ultra High-Speed Quantification of Cell Strain During Cell–Microbubble Interaction
Source: ACS Biomater Sci Eng. 2023 Sep 25;9(10):5912–23. doi: 10.1021/acsbiomaterials.3c00757 (PMC10565720; doi:10.1021/acsbiomaterials.3c00757)
Supplement: Supplementary file 1 — ab3c00757_si_001.zip [file ab3c00757_si_001.zip › Supplementary Information/Supporting Information.pdf]

Supporting Information for: An Acoustic Device for Ultra-high Speed Quantification of Cell Strain During Cell-Microbubble Interaction

Oliver Pattinson,[†] Sara Keller,[‡] Nicholas D. Evans,[†] Fabrice Pierron,[†] and Dario Carugo[*,¶]

† Department of Engineering, University of Southampton, University Road, SO17 1BJ, Southampton, UK

‡ Department of Engineering Science, University of Oxford, Old Road, Headington, OX3 7LD, Oxford, UK

¶ Nuffield Department of Orthopaedics, Rheumatology and Musculoskeletal Sciences (NDORMS), University of Oxford, Old Road, Headington, OX3 7LD, Oxford, UK

E-mail: dario.carugo@ndorms.ox.ac.uk

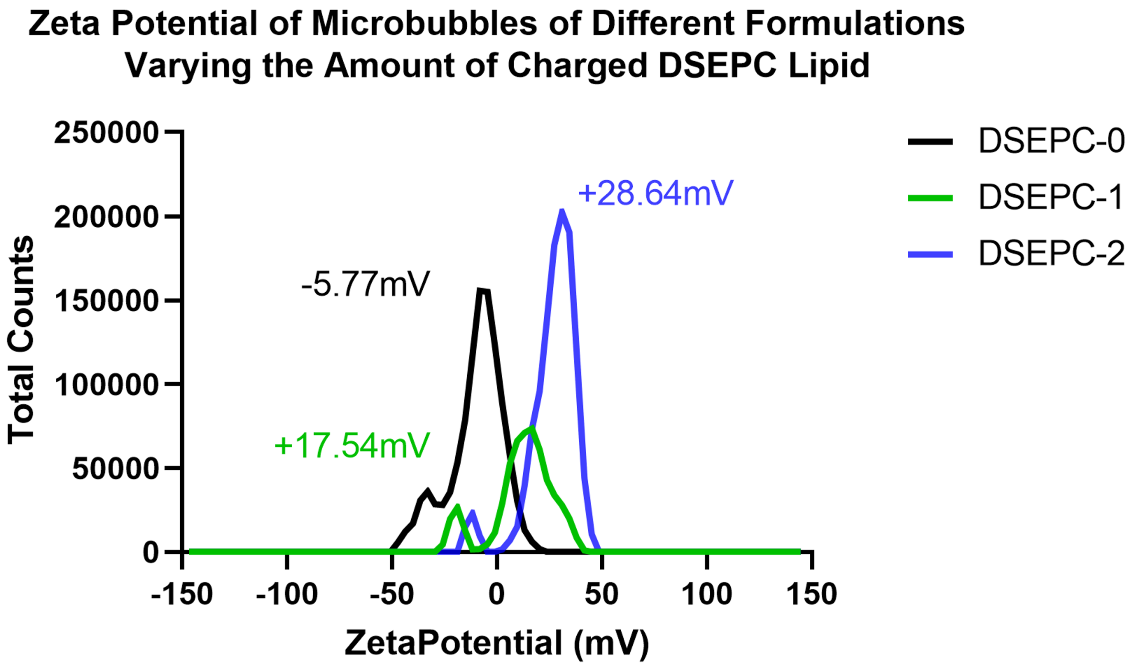

Figure 1: Zeta potential measurements for neutral DSPC-microbubbles and cationic DSEPC-microbubbles at two different molar concentrations measured using ZetaSizer Ultra (Malvern Panalytical Ltd., Worcester, UK) DLS machine.

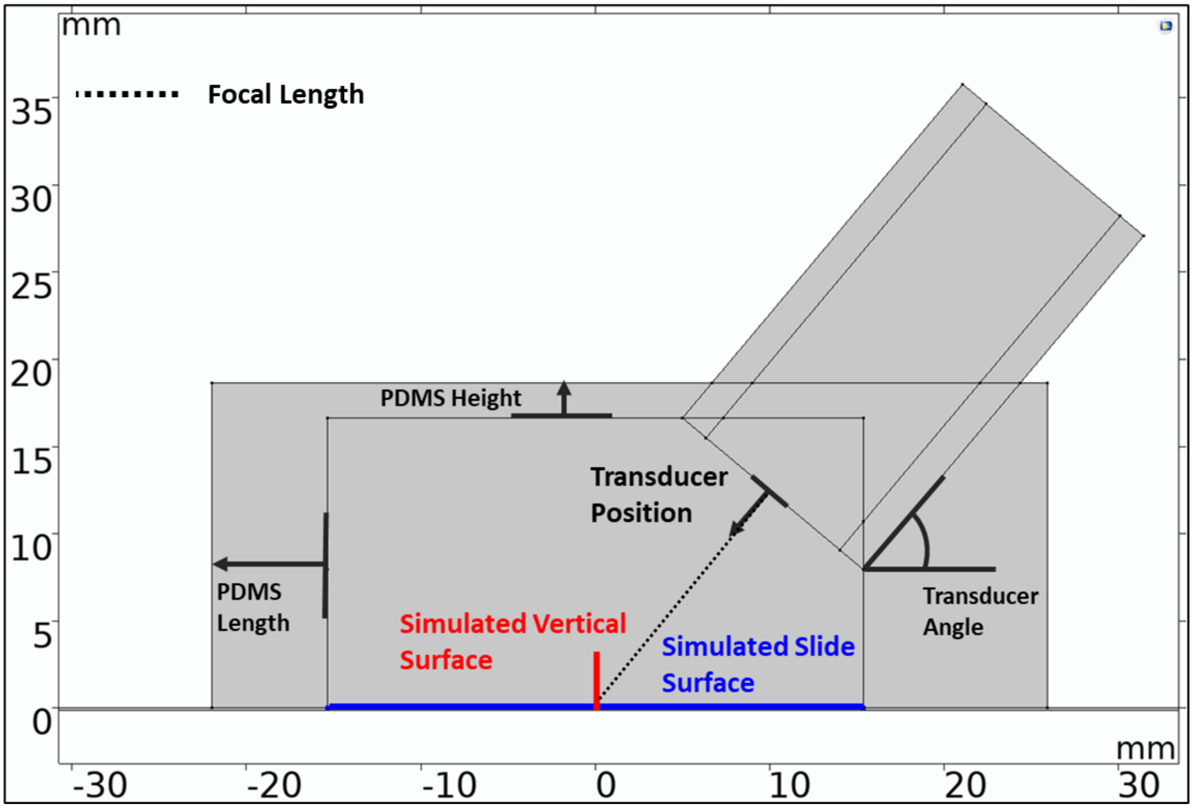

Figure 2: Acoustic device schematic with the inserted transducer. Reference geometry with labelled dimensions and areas of study for COMSOL Multiphysics 5.5 Pressure Acoustics simulations.

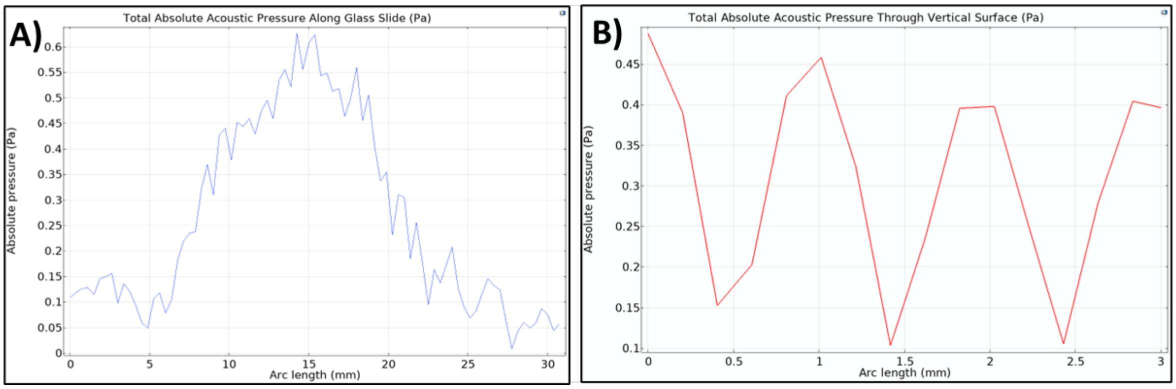

Figure 3: Simulated acoustic pressure profiles within the device with the finalised design parameters. A) The pressure profile in the horizontal x-direction along the glass slide B) The pressure profile in the vertical y-direction at the centre of the device.

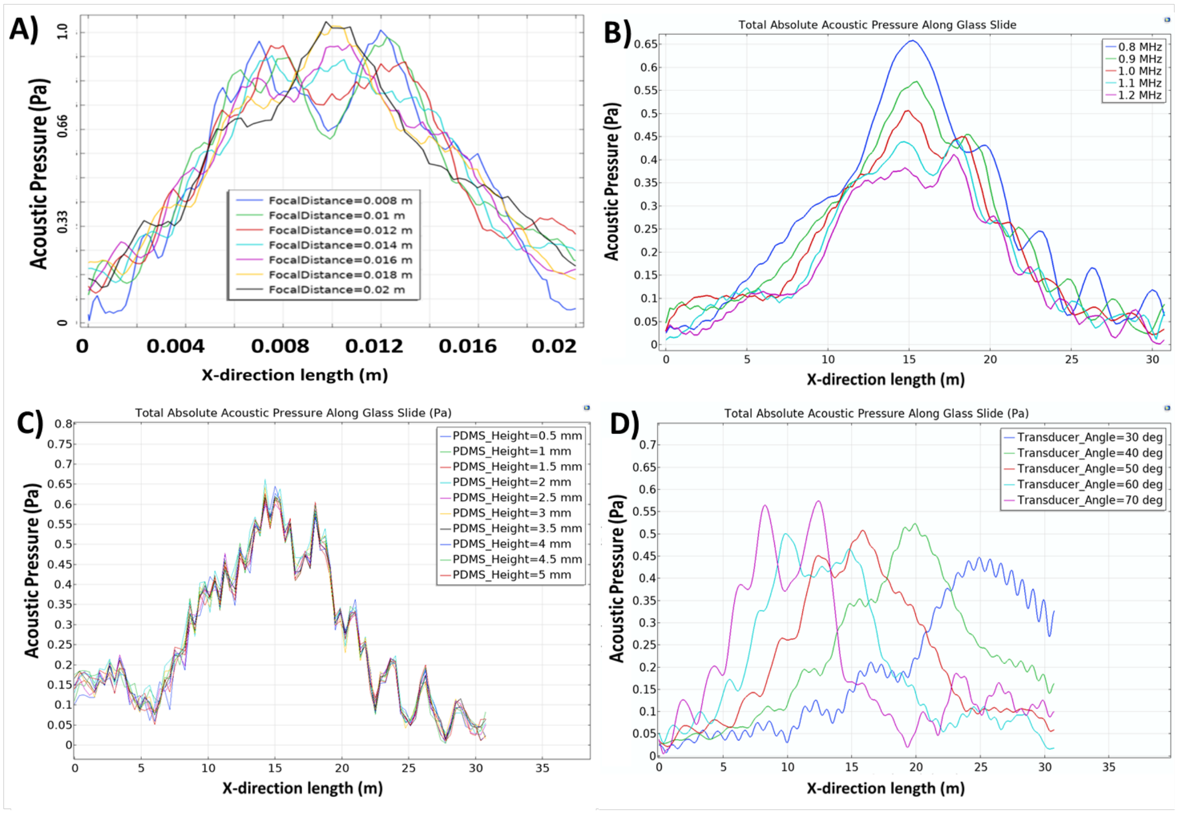

Figure 4: Simulated horizontal pressure profiles along the glass surface when design variables are varied. A) The effect of changing the focal distance. B) The effect of changing the transducer frequency. C) The effect of changing the PDMS Height. D) The effect of changing the transducer angle.

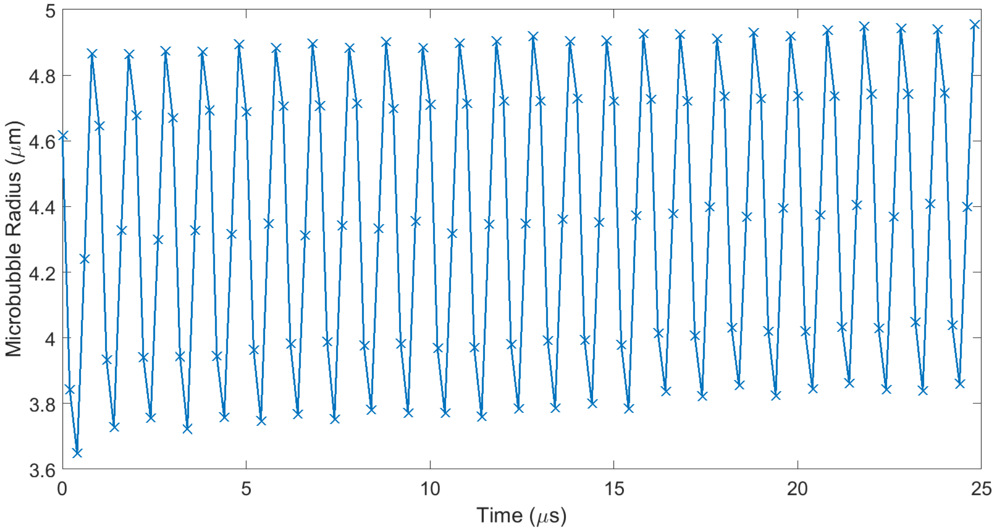

Figure 5: Extended graph showing the oscillation of a DSPC-microbubble interacting with an MG-63 cell, captured using UHS imaging at 5 million FPS over 128 frames, encompassing 25 μs.
